# Pancreatic cancer incidence trends in the United States from 2000-2017: Analysis of Surveillance, Epidemiology and End Results (SEER) database

**DOI:** 10.12688/f1000research.54390.1

**Published:** 2021-07-02

**Authors:** Hassam Ali, Rahul Pamarthy, Meghana Vallabhaneni, Shiza Sarfraz, Hadiqa Ali, Hamza Rafique

**Affiliations:** 1 Internal Medicine, East Carolina University, Greenville, North Carolina, 27834, USA; 2 Internal Medicine, University of Health Sciences, Lahore, Punjab, 42000, Pakistan

**Keywords:** Pancreatic cancer, SEER database, PNETs, pancreatic neuroendocrine tumors, pancreatic cancer risk factors, Pancreatic cancer incidence, SEER 18

## Abstract

**Background:** Recent incidence trends of pancreatic cancers were reviewed by demographics and histologic type to observe any new findings.

**Methods:** Data was used from the Surveillance, Epidemiology, and End Results (SEER) registry 18 (2000-2017) and it underwent temporal trend analysis. Pancreatic cancer incidence rates were reported based on histological subtype and demographics.

**Results:** The incidence rate of white males increased significantly during 2000-2017 (annual percent change (APC) = 3.5%) compared to previously reported APCs. The incidence of white females grew from an APC of 1.29% to 2.9%. Rates among black ethnicity increased with an APC of 4.2%. Rates among Hispanics and other ethnicities also showed increment. The rates for ductal adenocarcinoma showed a positive trend in all races, with the APC ≥ 6 % for females and APC ≥ 6.5 % for males. The rates of non-secretory endocrine tumors showed a decline in both genders of all five races in recent years after showing an initial positive trend till 2010. Rates for pancreatic adenocarcinoma continued to rise in all ethnicities from 2000-2017. Interestingly, there was a rise in carcinoid type pancreatic neuroendocrine tumors (PNETs) in all ethnicities. Cumulatively, males had a higher incidence than females; male to female Incidence Risk Ratio (IRRs) was 1.32. The IRR was > 1 for age groups ≥ 35 years. The male to female IRRs was less than 1 for cystic adenocarcinoma, secretory endocrine, and solid pseudopapillary carcinomas (IRR = 0.5, 0.9, and 0.2 respectively, confidence intervals 0.4–0.6 and 0.9-1.3, 0.2–0.3, respectively).

**Conclusion:** Pancreatic cancer incidence continued to rise in the years 2000-2017. However, incidence differed by demographics and histologic type. Interestingly, recent years discerned a rise in PNETs (carcinoid type) which has not been reported previously.

## Introduction

As per global reports in 2018, pancreatic cancer remains the twelfth most common cancer in men and the eleventh in women ^1^. Cancer-related deaths have pancreatic cancer as the seventh leading cause ^1^. In the United States of America (USA), pancreatic cancer (PC) incidence has been increasing as previously reported ^1, 2^. PC mortality is estimated to rise as time progresses, and recent projections estimate that it might overpass colorectal cancer to become the second leading cancer-related cause of death after lung cancer ^3^. In 2020 reports showed 57,600 new cases and 47,050 PC related deaths ^3^. The PC risk increases with age, and the median age of diagnosis is 71 years ^4^. Previously the increases were most significant for non-secretory endocrine cancers, followed by ductal adenocarcinomas and adenocarcinoma ^5^.

## Methods

The results were obtained from the analysis of the Surveillance Epidemiology and End Results (SEER) cancer registry database. SEER 18 includes 28% of the U.S. population as of the 2010 census ^6^. The rates are available by expanded race/ethnicity of cases diagnosed, including white, black, Asian/Pacific Islander, and American Indian/Alaskan Native and Hispanic ethnicity. SEER 18 also includes adjustments for areas impacted by hurricanes Katrina and Rita. It contains a record for each of 8,131,919 tumors. 19 age groups are available and can be classified as < 1 year, 1–4 years, 5–9 years to 85+ years, or unknown. The registries included in SEER 18 are San Francisco-Oakland SMSA, Connecticut, Detroit (Metropolitan), Hawaii, Iowa, New Mexico, Seattle (Puget Sound), Utah, Atlanta (Metropolitan, San Jose-Monterey, Los Angeles, Alaska Natives, Rural Georgia, California excluding San Francisco, San Jose Monterey, Los Angeles, Kentucky, Louisiana, New Jersey, Greater Georgia. SEER 18 encompasses the SEER 13, which itself includes seer 9 in addition to supplementary registries ^6^.

Within the SEER database, International Classification of Diseases (ICD)-0–2 was used through 2000 ^7^, and ICD-0–3 following 2001 for coding the primary site and histologic type of diagnosed pancreatic cancer cases ^8^. Site recode ICD-0–3/WHO 2008: Pancreas (ICD-O-3 site C25) was used via SEER*Stat software to select cases pancreatic cancer ^9^. Only cases that were malignant and had a microscopically confirmed diagnosis were included. The ages below 30 years were filtered out due to reportedly low incidence per previous literature ^5^. Cases were characterized into exclusive groups based on morphological classification of pancreatic cancer in the ICD (Numbers given below) for Oncology and previous similar study ^5, 8^. In addition to the undermentioned cases, all other cases were classified as poorly specified types.

• Adenocarcinoma, not otherwise specified (NOS) (8140)
• Mucinous adenocarcinoma (8480, 8481)
• Ductal adenocarcinoma excluding cystic or mucinous (8507, 8510, 8514, 8521, 8560, 8570, 8523, 8255, 8490, 8500)
• Cystic adenocarcinoma (8440, 8470, 8504)
• Intraductal papillary mucinous neoplasm (IPMN) (8453, 8471, 8503, 8144, 8450)
• Non-secretory endocrine (8150, 8246)
• Secretory endocrine (8151, 8152, 8156, 8153, 8155)
• Carcinoid tumors (8243, 8244, 8245, 8240, 8241)
• Acinar cell adenocarcinomas (8550, 8551),
• Solid pseudopapillary tumors (8452),
• Other adenocarcinoma (8145, 8260, 8441, 8154, 8574, 8460)
• Non-carcinomas (8680–9999)

SEER*Stat ^9^ was utilized to calculate the incidence and age-adjusted rates, grouped by histologic subtype, gender, ethnicity, and age group. SEER 18 registry was used for analyzing tends with detailed racial group categories from 2000 to 2017 using yearly diagnosis. The period-specific rates were plotted and utilized temporal trend figures at two-year intervals and utilized a logarithmic scale with a base of 10 degrees portrayed rate per 100,000 person-years ^10^. The annual percent changes (APCs) were quantified using annual rates for temporal trends ( Table 1 & Table 2), and 95% confidence intervals (CIs) were included. SEER*Stat ^9^ was used for all analyses ^6^. Incidence Rate Ratios (IRRs) based on gender and ethnicity/race were calculated; these were further classified based on age groups and histological types. 95% CIs for the IRRs was calculated using the Tiwari method, and the figures were generated using Microsoft excel. Potential sources of bias include concern of patient migration across seer registries. Data is de-identified by SEER, and this potential for sample bias cannot be intervened but mentioned in this study's limitations.

**Table 1.** Total cases and Annual Percentage Changes (APCs) of pancreatic cancer by race and gender (2000–2017).

Males				
Race	Count	APC	LCI	UCI
White	56991	3.4 *	3.3	3.6
Black	8807	4.2 *	3.8	4.6
American Indian/Alaska Native	396	7.4 *	4.7	10.1
Asian or Pacific Islander	5138	6.1 *	5.6	6.7
Hispanic (All Races)	7616	6.5 *	6	7
Non-Hispanic unknown races	133	17.1 *	12.2	22.2
Females				
Race	Count	APC	LCI	UCI
White	50344	2.9 *	2.7	3
Black	9489	4.2 *	3.8	4.6
American Indian/Alaska Native	383	2.7 *	0.7	4.7
Asian or Pacific Islander	5404	6.1 *	5.6	6.7
Hispanic (All Races)	7751	6.4 *	5.8	7
Non-Hispanic unknown races	~	~	~	~

APC = Annual percent change; UCI, LCI = Upper, Lower 95% confidence interval. Confidence intervals are 95% for trends. Percent changes were calculated using 1 year for each end point; APCs were calculated using weighted least squares method. ~ Statistic could not be calculated due to lower number of cases or at least one year with no reported cases. * The APC is significantly different from zero (p<0.05). Cases and annual percentage changes were only mentioned if there were enough cases to yield results after temporal trends analysis.

**Table 2.** Total cases and Annual Percentage Changes (APCs) of pancreatic cancer by race, gender, and age group (2000–2017).

Males				
Age groups	Count	APC	LCI	UCI
White				
30–34 years	112	3.3 *	0.2	6.4
35–39 years	359	0.2	-2.1	2.5
40–44 years	869	-2.3 *	-3.7	-0.9
45–49 years	1,989	-0.7	-1.5	0.2
50–54 years	3,931	0.9 *	0.2	1.6
55–59 years	6,201	3.1 *	2.3	4
60–64 years	7,997	4.7 *	4	5.4
65–69 years	9,271	5.7 *	5.1	6.4
70–74 years	8,978	3.8 *	3.1	4.5
75–79 years	7,959	2.4 *	1.8	2.9
80–84 years	5,871	2.7 *	1.9	3.4
85+ years	3,454	5.4 *	4.4	6.4
Black				
30–34 years	~	~	~	~
35–39 years	84	-1.3	-5.4	3
40–44 years	199	-0.5	-3	2
45–49 years	484	0.3	-1.6	2.3
50–54 years	945	2.0 *	0.1	3.9
55–59 years	1,333	4.4 *	2.9	6
60–64 years	1,534	7.0 *	5.8	8.3
65–69 years	1,384	4.9 *	3.6	6.2
70–74 years	1,180	4.3 *	3.1	5.5
75–79 years	847	4.0 *	2.2	5.9
80–84 years	505	2.8 *	1.3	4.4
85+ years	264	7.5 *	4.9	10.3
Asian or Pacific Islander				
30–34 years	~	~	~	~
35–39 years	~	~	~	~
40–44 years	125	2.2	-1.8	6.4
45–49 years	221	5.3 *	1.8	9
50–54 years	353	6.0 *	3.4	8.6
55–59 years	513	4.6 *	3.1	6.1
60–64 years	699	5.7 *	4.3	7.1
65–69 years	788	6.9 *	5.3	8.5
70–74 years	768	6.4 *	5.1	7.7
75–79 years	690	5.8 *	4	7.7
80–84 years	523	6.3 *	4.5	8.3
85+ years	353	7.5 *	5.2	9.9
Hispanics				
30–34 years	~	~	~	~
35–39 years	123	1.9	-2.4	6.4
40–44 years	252	5.7 *	2.8	8.6
45–49 years	454	3.9 *	1.5	6.4
50–54 years	745	6.3 *	5	7.6
55–59 years	914	7.2 *	5.6	8.8
60–64 years	1,152	7.9 *	6.6	9.2
65–69 years	1,119	7.3 *	6	8.6
70–74 years	1,090	5.5 *	4.1	6.9
75–79 years	866	5.3 *	3.9	6.7
80–84 years	545	6.3 *	3.9	8.8
85+ years	307	8.2 *	4.6	11.8
Females				
White				
30–34 years	138	0.8	-2.6	4.3
35–39 years	284	0.3	-2.4	3.1
40–44 years	672	0	-1.6	1.6
45–49 years	1,427	-0.2	-1.6	1.3
50–54 years	2,697	1.6 *	0.6	2.6
55–59 years	4,402	3.6 *	2.6	4.6
60–64 years	5,793	4.7 *	4.1	5.4
65–69 years	7,150	5.0 *	4.3	5.8
70–74 years	7,806	2.5 *	1.7	3.2
75–79 years	8,183	1.3 *	0.8	1.7
80–84 years	6,672	1.7 *	0.9	2.5
85+ years	5,120	4.2 *	3.6	4.8
Black				
30–34 years	~	~	~	~
35–39 years	~	~	~	~
40–44 years	194	-0.2	-3.3	3.1
45–49 years	454	1.4	-0.4	3.3
50–54 years	734	3.1 *	1.3	4.8
55–59 years	1,043	6.3 *	4.7	7.9
60–64 years	1,386	6.0 *	4.7	7.2
65–69 years	1,478	5.3 *	3.7	6.9
70–74 years	1,353	3.6 *	2.5	4.6
75–79 years	1,205	3.2 *	2.2	4.3
80–84 years	899	3.4 *	2.1	4.7
85+ years	602	3.6 *	1.3	5.9
Asian or Pacific Islander				
30–34 years	~	~	~	~
35–39 years	~	~	~	~
40–44 years	110	3.0 *	0	6
45–49 years	186	4.0 *	0.7	7.4
50–54 years	286	2.8	-1.1	6.9
55–59 years	474	5.6 *	3.9	7.4
60–64 years	625	7.1 *	5.5	8.7
65–69 years	775	5.8 *	4.7	7
70–74 years	866	5.2 *	3.8	6.6
75–79 years	861	5.8 *	4	7.7
80–84 years	662	6.2 *	4	8.5
85+ years	475	9.7 *	7.7	11.8
Hispanic				
30–34 years	~	~	~	~
35–39 years	118	6.5 *	2.7	10.5
40–44 years	206	6.2 *	4	8.5
45–49 years	387	6.1 *	3.9	8.3
50–54 years	563	6.4 *	4.4	8.4
55–59 years	808	6.3 *	4.3	8.3
60–64 years	1,035	7.2 *	5.7	8.7
65–69 years	1,112	7.0 *	5.5	8.6
70–74 years	1,133	4.9 *	3.8	6
75–79 years	1,080	4.7 *	3.7	5.7
80–84 years	722	7.8 *	6.6	9
85+ years	515	6.8 *	4.7	8.9
Males				
Histological Types	Count	APC	LCI	UCI
White				
Adenocarcinoma, not otherwise specified	39,237	3.3 *	3	3.5
Ductal adenocarcinoma excluding cystic or mucinous	6,292	6.6 *	6	7.3
Ductal specified as arising from an intraductal papillary mucinous neoplasm	195	4.6 *	2	7.3
Non-secretory Endocrine	2,572	3.8 *	0.7	7.1
Secretory endocrine	67	1.6	-2.2	5.6
Carcinoid tumors	1,244	32.5 *	26.8	38.4
Acinar cell adenocarcinomas	242	5.9 *	3.1	8.7
Mucinous adenocarcinoma	2,079	-3.0 *	-3.9	-2.1
Other adenocarcinoma	170	-0.4	-3	2.3
Black				
Adenocarcinoma, not otherwise specified	6,203	4.2 *	3.7	4.7
Ductal adenocarcinoma excluding cystic or mucinous	859	7.5 *	6.5	8.6
Endocrine: non-secretory	328	6.1 *	1.1	11.3
Mucinous adenocarcinoma	274	-2.7 *	-4.9	-0.5
American Indian/Alaska Native				
Adenocarcinoma, not otherwise specified	281	7.0 *	3.9	10.2
Ductal adenocarcinoma excluding cystic or mucinous	40	13.4 *	8.4	18.7
Asian or Pacific Islander				
Adenocarcinoma, not otherwise specified	3,334	5.7 *	4.8	6.5
Ductal adenocarcinoma excluding cystic or mucinous	649	8.5 *	6.6	10.4
Endocrine: non-secretory	237	6.7 *	2	11.5
Mucinous adenocarcinoma	183	2.1	-1.3	5.5
Hispanics				
Adenocarcinoma, not otherwise specified	5,208	6.1 *	5.4	6.7
Ductal adenocarcinoma excluding cystic or mucinous	807	11.3 *	9.6	13
Endocrine: non-secretory	328	6.8 *	2.9	10.8
Mucinous adenocarcinoma	237	2	-0.4	4.5
Females				
White				
Adenocarcinoma, not otherwise specified	34,955	2.9 *	2.6	3.1
Ductal adenocarcinoma excluding cystic or mucinous	5,736	6.1 *	5.5	6.8
Cystic adenocarcinoma	185	-9.1 *	-12.1	-6.1
Ductal specified as arising from an intraductal papillary mucinous neoplasm	165	3.4 *	0.9	5.9
Endocrine: non-secretory	1,785	3.7 *	0.5	7
Secretory endocrine	80	-1.8	-5	1.6
Carcinoid tumors	863	30.3 *	24.5	36.4
Acinar cell adenocarcinomas	82	2.1	-1.3	5.7
Mucinous adenocarcinoma	2,032	-2.7 *	-3.7	-1.6
Other adenocarcinoma	130	-1.1	-4.9	2.9
Black				
Adenocarcinoma, not otherwise specified	6,615	4.0 *	3.5	4.4
Ductal adenocarcinoma excluding cystic or mucinous	961	8.4 *	6.8	10
Endocrine: non-secretory	363	3.7	-0.2	7.8
Carcinoid tumors	228	26.9 *	20.9	33.2
Mucinous adenocarcinoma	355	-2.8 *	-4.7	-0.9
American Indian/Alaska Native				
Adenocarcinoma, not otherwise specified	260	1.1	-1.2	3.5
Ductal adenocarcinoma excluding cystic or mucinous	43	7.1 *	3.9	10.5
Asian or Pacific Islander				
Adenocarcinoma, not otherwise specified	3,552	5.7 *	5	6.4
Ductal adenocarcinoma excluding cystic or mucinous	746	9.6 *	7.5	11.7
Endocrine: non-secretory	196	4.6	-0.9	10.4
Mucinous adenocarcinoma	195	0.1	-3.2	3.5
Hispanic				
Adenocarcinoma, not otherwise specified	5,148	6.0 *	5.3	6.7
Ductal adenocarcinoma excluding cystic or mucinous	872	10.1 *	8.2	12
Endocrine: non-secretory	315	6.5 *	2.7	10.6
Mucinous adenocarcinoma	307	-0.4	-2.5	1.7

APC = Annual percent change; UCI, LCI = Upper, Lower 95% confidence interval. Confidence intervals are 95% for trends. Percent changes were calculated using 1 year for each end point; APCs were calculated using weighted least squares method. ~ Statistic could not be calculated due to lower number of cases or at least one year with no reported cases. * The APC is significantly different from zero (p<0.05). Cases and annual percentage changes were only mentioned if there were enough cases to yield results after temporal trends analysis.

## Results

### Temporal trends

**Figure 1.**
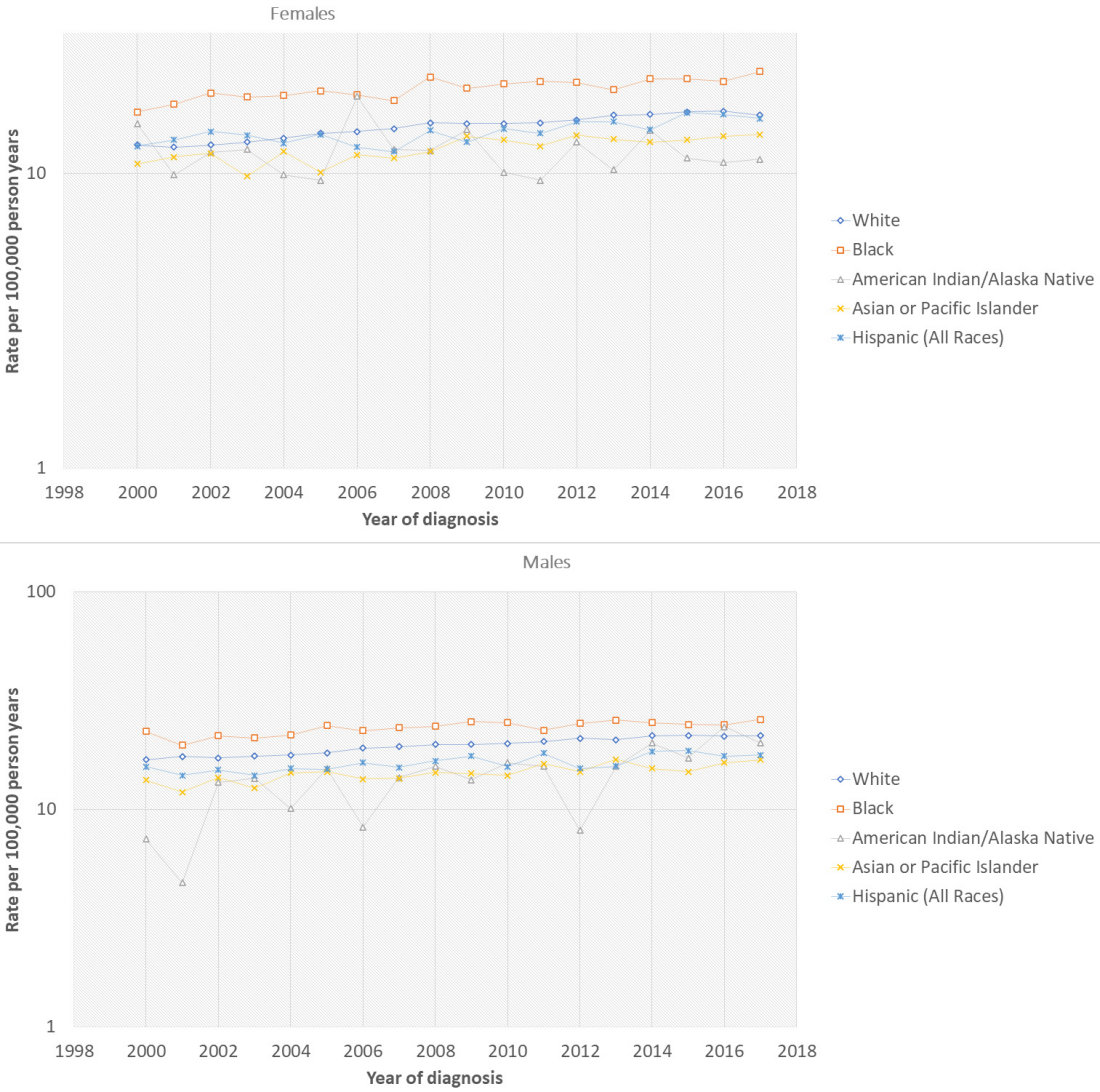
Pancreatic cancer incidence trends by gender and race based on SEER 18 (2000–2017). a: Females, b: Males.

SEER 18 allowed us to explore short-term, nonetheless, relatively recent and previously unreported trends in pancreatic cancer ( Table 1) ( Figure 1). The incidence rate of white males has increased significantly during 2000–2017 (APC = 3.5%) compared to previously reported APC of 0.95% from 1994–2013 ^5^. The incidence of white females also increased from an APC of 1.29% (1999–2013) to 2.9% (2000–2017) ^5^. Rates among black males and females have also increased in recent years, per our analysis, with an APC of 4.2% each, respectively. Previously, the rates among black males decreased, while rates remained unchanged among black females (1975–2013) ^5^.

Rates among Hispanic men and women have continued to increase (APC = 6.5% and 6.4%). Similarly, rates among American Indian/Alaska Native in men increased more than women (APC = 7.4% vs. 2.1%). In Asian or Pacific Islander, the APC was similar in both genders (6.1%) as compared to lower incidence rates of 0.16% for males and 1.23% for females (1992–2013) ^5^.

**Figure 2.**
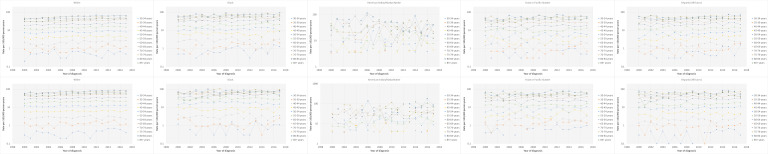
Pancreatic cancer incidence trends by age groups, gender and race based on SEER 18 (2000–2017). a: Males, b: Females.

There was a positive trend in rates for white males from ages 30–39 and ≥50 years for the 5-year age groups up to 85 and above as shown in Table 2 and Figure 2. White females showed a statistically significant positive trend from age ≥50. Interestingly, ages 40–49 showed a negative trend in white males, and age group 40–44 had a statistically significant negative APC of -2.3%.

Both genders in the black population showed a positive trend after age ≥ 45 ( Table 2) ( Figure 2). It was impossible to calculate APCs among younger age groups for black females due to at least one year with zero cases. Age-specific rates also rose among Asians or pacific islanders and Hispanic men from ages ≥ 35 and ≥ 40, respectively ( Figure 2). APCs for females of Asians or pacific islanders and Hispanic ethnicity also showed a positive trend after ages ≥ 40 and ≥ 35, respectively ( Figure 2). Further analysis was performed to explore trends in American Indian/Alaskan native ( Figure 2). However, APCs could not be calculated in either males or females for any age group due to at least one year with zero cases. The results are therefore not shown in tables. From 2000 to 2017, for ages, more than 30 years, 152,548 of 190,586 pancreatic cancer cases (80.04%) diagnosed had a microscopic confirmation. This represented an increase compared with the 77% microscopically confirmed cases from 1992–2013, including all ages. (Results are not shown). We did not report the analysis of the temporal trends by following histologic-type groups due to the number of cases being too low: cystic adenocarcinoma (n= 408), ductal specified as arising from an IPMN (n = 520), secretory endocrine (n = 203), other adenocarcinomas: acinar cell (n = 438), solid pseudopapillary tumors (n = 228), other adenocarcinomas (n = 448), other non-carcinomas (n = 6).

**Figure 3.**
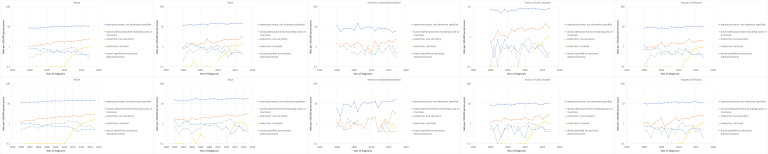
Pancreatic cancer incidence trends by age groups, gender and histological type based on SEER 18 (2000–2017). a: Males, b: Females.

Rates for pancreatic adenocarcinoma, the most common histologic type of pancreatic cancer, continued to rise in all ethnicities from 2000–2017. The rates for ductal adenocarcinoma (excluding mucinous and cystic) showed a positive trend in all races (including American Indian/Alaska Native) with the APC ≥ 6 % for females and APC ≥ 6.5 % for males ( Table 2) ( Figure 3). The rates of non-secretory endocrine tumors showed a decline in both genders of all five races in recent years after showing an initial positive trend till 2010. However, the APC from 2000–2017 remained positive and ≥ 3.5% for males and females ( Table 2). APC for American Indian/Alaska Native was not calculated due to at least one year with zero cases; however, trends are reported in Figure 3.

Interestingly, the rates for carcinoid endocrine tumors showed a positive trend and rise in rates from 2010 and onwards ( Figure 3), which was not reported previously. The APC was highest in white males and females, and black females (32.5%, 30.3%, and 26.9%, respectively). APCs for the rest of the races were not reported as statistics were incalculable as at least one year had no reported cases from 2000 to 2017.

### Male to female IRRs

In SEER 18 (2000–2017), 152,548 cases were microscopically confirmed from ages ≥ 30; males: 79081 and females: 73,467. This was higher than previously reported rates, which was 69,049 in males and 69,548 in females (2000–2013) ^5^. The IRR was greater in males’ total cases (IRR 1.32,95% CI 1.30-1.33) ( Table 3). All age groups from 35 and above showed an increased incidence risk in males vs. females (IRRs >1: increased risk) ( Table 3). Rates among males were higher compared to females for all histological subtypes except ductal cystic adenocarcinoma (0.5,95% CI 0.4-0.6), secretory endocrine tumors (0.9, 95% CI 0.9-1.3), solid pseudopapillary (0.2, 95% CI 0.2-0.3) and non-carcinomas (0.1, 95% CI 0.7-7.7). Carcinoid endocrine tumors also showed a greater IRR in males (1.4, 95% CI 1.3-1.5) ( Table 3).

**Table 3.** Total cases, rates, and male to female incidence rate ratios, by age group and histologic type (2000–2017).

Males and Females			Males			Females			Male/Female		
	Count	% of total	Count	% of total	Rate	Count	% of total	Rate	IRR	LCI	UCI
Total cases	152548	100	79081	51.84	19.4	73467	48.15	14.7	1.32	1.30	1.33
Age groups											
30–34 years	543	0.35	243	0.3	0.5	300	0.4	0.6	0.8	0.67	0.95
35–39 years	1,197	0.78	646	0.8	1.2	551	0.7	1	1.2	1.04	1.31
40–44 years	2,651	1.7	1,458	1.8	2.7	1,193	1.6	2.2	1.2	1.14	1.33
45–49 years	5,668	3.7	3,182	4.0	5.9	2,486	3.3	4.5	1.3	1.24	1.38
50–54 years	10,364	6.7	6,041	7.6	11.9	4,323	5.8	8.2	1.5	1.39	1.50
55–59 years	15,815	10.3	9,025	11.4	20.4	6,790	9.2	14.5	1.4	1.37	1.45
60–64 years	20,378	13.3	11,465	14.49	32.5	8,913	12.1	23.1	1.4	1.37	1.44
65–69 years	23,226	15.2	12,645	15.98	46.8	10,581	14.4	34.4	1.4	1.32	1.39
70–74 years	23,319	15.2	12,082	15.27	60	11,237	15.3	46.1	1.3	1.27	1.34
75–79 years	21,789	14.2	10,418	13.17	69.4	11,371	15.5	56.9	1.2	1.19	1.25
80–84 years	16,467	10.7	7,476	9.4	72.5	8,991	12.2	57.4	1.3	1.23	1.30
85+ years	11,131	7.2	4,400	5.5	53.8	6,731	9.1	40.5	1.3	1.28	1.38
Histological type											
Total	139267	100	72097	51.76	17.6	67170	48.23	13.5	1.31	1.29	1.32
Adenocarcinoma, not otherwise specified	104,926	75.3	54344	75.4	13.3	50582	75.3	10.1	1.3	1.30	1.34
Ductal adenocarcinoma excluding cystic or mucinous	17,026	12.2	8658	12	2.1	8368	12.5	1.7	1.25	1.21	1.3
Cystic adenocarcinoma	408	0.2	118	0.1	0.0	290	0.4	0.1	0.5	0.4	0.6
Ductal specified as arising from an intraductal papillary mucinous neoplasm	520	0.3	268	0.3	0.1	258	0.3	0.1	1.3	1.1	1.5
Endocrine: non-secretory	6,167	4.4	3484	4.8	0.8	2683	3.9	0.6	1.5	1.4	1.6
Secretory endocrine	203	0.1	94	0.1	0.0	109	0.1	0.0	0.9	0.9	1.3
Carcinoid tumors	3,201	2.2	1753	2.4	0.4	1448	2.1	0.3	1.4	1.3	1.5
Acinar cell adenocarcinomas	438	0.3	314	0.4	0.1	124	0.2	0.0	3.0	2.5	3.8
Mucinous adenocarcinoma	5,696	4.1	2790	3.9	0.7	2906	4.3	0.6	1.2	1.1	1.2
Solid pseudopapillary tumors	228	0.2	42	0.05	0.0	186	0.3	0.0	0.2	0.2	0.3
Other adenocarcinoma	448	0.3	229	0.3	0.1	219	0.3	0.1	1.3	1.1	1.5
Other non-carcinomas	6	0.004	3	0.004	0.0	3	0.004	0.0	0.9	0.1	7.7

Rates are per 100 000 person-years, age-adjusted to the 2000 US Standard Population (19 age groups - Census P25–1130). IRR = Incidence Rate Ratios (based on unrounded rates); LCI, UCI = lower, upper 95% confidence interval. Confidence intervals (Tiwari mod) are 95% for the ratios.

### Incidence risk ratios IRRs by racial/ethnic group

For SEER 18 (2000–17), the highest rates for pancreatic cancer in ages greater than 30, were in both black males and females (IRR = 1.21 and 1.36, respectively) when compared to white (non-Hispanic) ethnicity. Incidence, when compared to white (non-Hispanic) ethnicity, was higher in both black males and females (IRR = 1.21 and 1.36, respectively). The rates for both genders in American Indian/Alaska, Native Asian, or Pacific Islander Hispanic (All Races) was lower as compared to the white(non-Hispanic) race ( Table 4).

**Table 4.** Total cases, rates, and male to female incidence rate ratios by gender and racial/ethnic group, (2000–2017).

Males						Females				
Race	Count	Rate	IRR	LCI	UCI	Count	Rate	IRR	LCI	UCI
White	56,991	19.8	0	0	0	50,344	14.5	0	0	0
Black	8,807	23.9	1.21	1.18	1.24	9,489	19.6	1.36	1.33	1.39
American Indian/Alaska Native	396	14.9	0.75	0.67	0.84	383	11.8	0.82	0.74	0.91
Asian or Pacific Islander	5,138	14.9	0.76	0.73	0.78	5,404	12.4	0.85	0.83	0.88
Hispanic (All Races)	7,616	16.6	0.84	0.82	0.86	7,751	14.1	0.97	0.95	0.99

Rates are per 100 000 person-years, age-adjusted to the 2000 US Standard Population (19 age groups - Census P25–1130). IRR = Incidence Rate Ratios (based on unrounded rates); LCI, UCI = lower, upper 95% confidence interval. Confidence intervals (Tiwari mod) are 95% for the ratios. The reference group is White non-Hispanic.

## Discussion

Our statistical analysis for SEER 18 registry revealed reportable findings by histologic subtype and demographic details. Incidence rates of pancreatic carcinomas increased amongst white males and females consistently. Incidence rates for black, Asian, or pacific inlander and Hispanics all rose in recent years for both genders. Rates for American Indian/Alaskan natives were inconsistent throughout 2000–2017; however, the final annual percent change remained positive for males (7.4%, CI 4.7-10.1) and females (2.7%, 0.7-4.7). Based on histological type, the overall increase in incidence remained secondary to pancreatic adenocarcinoma, as mentioned in a previous similar study ^5^.

Pancreatic carcinoma rates more predominantly rose in males when compared to females. In white males and females, there was a female prominence noted for incidence rates of age group 30–34 ( Table 2, Table 3). Rates for other ethnicities for ages <45 were comparable or could not be analyzed due to the lower number of cases. Rates of common histologic types were more in males, except for cystic adenocarcinoma, secretory endocrine cancers, solid pseudopapillary cancers, and non-carcinomas. This differs based on the histological type could be attributed to cystic adenocarcinoma and solid pseudopapillary cancers as they had the highest female predominance. Previous research comparing male to female incidence rate ratios also came to a similar conclusion for long-term rates from 1975–2003 and 2000 – 2013 ^5^. A review of 718 patients in 2005 stated a female predominance of solid pseudopapillary pancreatic cancers; almost 90% occurred in younger females aged between 19 to 50, with a ratio of close to 10:1 for females to males ^11^. A systemic review also showed an increased predominance of cystic adenocarcinomas in females, and our results were consistent with these studies ^12^.

An interesting finding in our analysis was the recent rise in pancreatic endocrine carcinoid tumors in all ethnicities. There was a male predominance with (IRR 1.4%, CI 1.3-1.5). The rates were highest in white males and started to increase after 2010 in all races except American Indian/Alaska Native who showed sporadic rise ( Figure 3). The black and Asian or pacific inlanders' incidence rates showed some plateau/decline in recent years ( Figure 3). A previous study on pancreatic neuroendocrine tumors (PNETs) suggested that cigarette smoking, Type II Diabetes, and family history in a first-degree relative are independent risk factors for non-functional PNETs. Another independent risk factor for functional PNETs was found to be heavier consumption of alcohol ^13^. No study currently comments solely on the risk factors of pancreatic carcinoid tumors, and it would be interesting to see associated risk factors for this recent rise. Our analysis revealed a rising rate of pancreatic carcinoid tumors with age, however, the results declined for ages >75 (results not shown).

Rates for intraductal papillary mucinous tumors and secretory endocrine carcinomas were similar in both genders. IPMN are malignancies of the pancreas that grow within the pancreatic ducts and produce mucin, hence the name. The risk factors of IPMN are not well understood. However, possible risk factors include insulin-dependent diabetes mellitus, chronic pancreatitis, positive family history, and smoking ^14^. Our analysis revealed a decline in mucinous adenocarcinomas ( Figure 3). This is consistent with the previously reported study ^5^. This can be explained by the fact that improved diagnostic imaging has enabled the detection of cystic or mucinous precursor lesions at an earlier stage. Our analysis only included only malignant lesions ^15^.

Numerous risk factors exist for pancreatic carcinoma and could be the reason behind the rising incidence. Individuals with non-O blood groups (type A, AB, or B) are more prone to the risk of pancreatic cancer ^16^. A possible link between Helicobacter pylori infection and non-O blood type may also exist; however, evidence is still lacking ^17^. Cystic fibrosis patients are at a greater risk of pancreatic cancer, especially adenocarcinoma, as their life span increases. It was also two to five-fold higher in patients with a history of organ transplantation ^18^. Up to 10% of pancreatic cancer patients have a family history ^19^. Some studies, though not explicit, identified a germline mutation involving a known susceptibility gene for pancreatic cancer (including BRCA, ATM, CDKN2A and PALB2) in patients with familial etiology ^20^.

 Chronic pancreatitis is also a risk factor for pancreatic cancer ^21^. This is hypothesized due to inflammation-induced metaplasia of the acinar cells into ductal cells by NF-kB and matrix metalloproteinases ^21^. Patients with a type of neoplastic pancreatic cyst, intraductal papillary mucinous neoplasm of the pancreas (IPMN) can develop an invasive malignancy called IPMN-associated adenocarcinoma. These patients can also develop ductal adenocarcinoma ^22^. Smoking is the most common cause of pancreatic cancer. Studies show a linear relationship with the number of cigarettes smoked ^23^. However, the risk of pancreatic cancer decreases by 48% two years after smoking cessation. This is equivalent to that of non-smokers by 10 to 15 years after smoking cessation ^23^. Individuals who struggle with body weight develop pancreatic cancer at a relatively younger age with shorter survival compared to individuals with normal bodyweight ^24^. Data regarding the effects of alcohol and coffee consumption has always been conflicting. Pooled analysis data showed a slight increase in the risk of pancreatic cancer in heavy drinkers and a high intake of coffee. This could be due to the confounder, which is cigarette smoking ^25^. An inverse relationship between consumption of a healthy diet and pancreatic cancer ^26^. However, the protective advantages of a healthy diet were only significant in men who were overweight/obese ^26^. Low levels of selenium and lycopene were found in subjects who developed pancreatic cancer in later life, however, the results are unclear ^27^. Though studies have shown an association with Hepatitis B and C with pancreatic cancer, the magnitude of risk is still significantly less than hepatocellular cancer ^28^. Numerous studies show an association between diabetes and pancreatic cancer. A meta-analysis showed the relative risk for pancreatic cancer in diabetes compared to the non-diabetic counterparts was 2.08 ^29^. Defects in metabolism of glucose, insulin resistance, and hyperinsulinemia are few etiologic factors for pancreatic cancer ^30– 32^. Increased risk of pancreatic cancer in patients with metabolic syndrome could be due to lower levels of adiponectin (a protein hormone linked to glucose level regulation and fatty acid breakdown), which results in a lack of insulin-sensitizing and anti-inflammatory capabilities ^33^.

Our study had its limitations, especially from an etiological point of view, as SEER does not report any risk factors, which can influence these trends. Comorbidities like obesity, diabetes, smoking are also not reported. There is a concern of migrating patients in and out of areas registered in the SEER database and selection bias. The data of the SEER registry holds high-grade quality, and statistics based on population can be informative of risk patterns and trends based on time. The critical strength of our studies was the large sample size, as SEER 18 covers more recent cases up to 2017 and covers almost 30% of the US population. It also allowed us to analyze histological types of pancreatic cancer.

## Conclusion

Pancreatic cancer incidence rates have continued to rise according to SEER 18 (2000–2017) in the USA. Our analysis concludes incidence of pancreatic cancer patterns based on demographical data and histologic type. We report the new rising incidence of pancreatic carcinoid tumors and the continued rise of pancreatic adenocarcinoma. We also report an increased incidence of cystic adenocarcinoma and solid pseudopapillary cancers in females as compared to males. These trends could be secondary to known risk factors for pancreatic carcinomas. Further comparative data regarding exclusive risk factors and contributing factors for histological subtypes of pancreatic carcinomas, specially PNETs, are required to understand the recent increase in incidence.

## Data availability

All data underlying the results are available as part of the article and no additional source data are required.

## Author contributions

**Hassam Ali:** Conceptualization, Methodology, Software, Validation, Writing- Reviewing and Editing. **Meghana Vallabhaneni**: Data curation, Writing- Original draft preparation. **Rahul Pamarthy:** Visualization, Project administration, Investigation. **Shiza Sarfraz**: Supervision, software, resources. **Hadiqa Ali**: Software, Validation. **Hamza Rafique**: Writing- Reviewing and Editing.
