# Supplementary material for: Pancreatic cancer incidence trends in the United States from 2000-2017: Analysis of Surveillance, Epidemiology and End Results (SEER) database
Source: F1000Res. 2021 Jul 2;10:529. [Version 1] doi: 10.12688/f1000research.54390.1 (PMC8411275; doi:10.12688/f1000research.54390.1)
Supplement: Peer review history

# Peer review: Reviewer response for version 1

**DOI:** 10.5256/f1000research.57873.r88966

**Published:** 2021-09-01

**Authors:** Harigopal Sandhyavenu

**Affiliations:** 1 Department of Cardiovascular Medicine, Mayo Clinic, Rochester, MN, USA

An interesting way of looking at the temporal trends of Pancreatic cancers. It is surprising to see increasing in the overall incidence, especially pancreatic endocrine carcinoid tumors among all ethnicities. Is it an actual rise in their number, or increasing positive tests with advanced diagnostic strategies? Either way this paper will allow the readers to look for possible correlation, if any. Overall this is an interesting article.

Is the work clearly and accurately presented and does it cite the current literature?

Yes

If applicable, is the statistical analysis and its interpretation appropriate?

Yes

Are all the source data underlying the results available to ensure full reproducibility?

Yes

Is the study design appropriate and is the work technically sound?

Yes

Are the conclusions drawn adequately supported by the results?

Yes

Are sufficient details of methods and analysis provided to allow replication by others?

Yes

Reviewer Expertise:

General Internal Medicine, Cardiology, Geriatrics

I confirm that I have read this submission and believe that I have an appropriate level of expertise to confirm that it is of an acceptable scientific standard.

# Peer review: Reviewer response for version 1

**DOI:** 10.5256/f1000research.57873.r88964

**Published:** 2021-07-12

**Authors:** Syed Hamza Bin Waqar

**Affiliations:** 1 Department of Medicine, State University of New York, Downstate Health Sciences University, Brooklyn, NY, USA

Interesting article which is dealing with SEER database to study one of the most invasive pancreatic CA out there. I like the design of the study and it's a smart idea to build up a database from SEER. It is interesting to note that PNETs are now going up the trend, although the reason is uncertain. It might be something that scientists/doctors would like to look into moving forward - as to why the incidence is increasing now? Is it just coincidence or is there is an actual correlation and if so, with what? A very thorough epidemiological, smart, and short study.

Is the work clearly and accurately presented and does it cite the current literature?

Yes

If applicable, is the statistical analysis and its interpretation appropriate?

Yes

Are all the source data underlying the results available to ensure full reproducibility?

Yes

Is the study design appropriate and is the work technically sound?

Yes

Are the conclusions drawn adequately supported by the results?

Yes

Are sufficient details of methods and analysis provided to allow replication by others?

Yes

Reviewer Expertise:

Gastroenterology, Hematology-Oncology

I confirm that I have read this submission and believe that I have an appropriate level of expertise to confirm that it is of an acceptable scientific standard.
